# Synthesis and research of epoxy resin toughening agent

**DOI:** 10.1186/s40064-016-2356-5

**Published:** 2016-06-21

**Authors:** Hongming Ma, Nannan Hu, Cheng Wu, Yinfeng Zhu, Yi Cao, Qing Qing Chen

**Affiliations:** 1 Power Research Institute of Yunnan Power Grid Co., Ltd., Kunming, 650217 China; 2 Institute of Plasma Physics, Chinese Academy of Sciences, Hefei, 230031 China; 3 School of Mechanical and Electrical Engineering, Anhui Jianzhu University, Hefei, 230601 China; 4 Yin Sudan Electric Co., Ltd., Hefei, 230031 China

**Keywords:** Epoxy resin, Toughening agent, Bonding strength, Two-phase structure

## Abstract

In this paper, a synthesis method of epoxy resin toughening agent was presented, then the chemical composition and molecular number were studied, which include the DSC curves analysis, the fracture surface morphology and bonding strength. In addition, the mechanism of epoxy resin toughening agent and the effect of toughening agent’s content to bonding strength were studied. The testing results reveal that this toughening agent can form a micro two-phase structure in epoxy-amine system, which results in the stable chemical properties and excellent physical properties.

## Background

Due to excellent mechanical, electrical properties and chemical stability, epoxy resin (EP) curing products are widely used in electronics, machinery, construction and other industries (FInk 2013). However, the brittleness of EP affects its further application. To ensure impact resistance and reduce the stress during curing, some measures should be taken to improve the toughening of EP curing products.

Generally, the EP toughening methods mainly include thermoplastic resin toughening, rubber toughening, organic silicon resin toughening, rigid particle toughening and nano-particles toughening, etc. Rubber toughening mechanism is the “silver streaks—nail anchor” mechanism and “silver lines—shear zone” mechanism (Yahyaie and Ebrahimi 2013). After having toughened with rubber, the resistance to impact and bending performances have been significantly improved (Rahman et al. 2013), however, the strength, modulus and heat resistance performance is weakened due to the lower strength and modulus of rubber. Thermoplastic resin modified by rubber can improve the toughness (Giannotti et al. 2003), but the stiffness and heat resistance are reduced. Nano-particles have large surface activity, which is easy to produce physical or chemical combination with the polymer (Al-Turaif 2010), which can resist micro-crack initiation. Compared with EP composite material toughened by rubber, the strength and rigidity are decreased (Créac’hcadec et al. 2014).

To obtain high performance of EP composite material, some new toughening methods and techniques are proposed, such as the macromolecular curing agent toughening, hollow particle toughening (Tagliavia et al. 2011) and resin alloying toughening. This paper describes a new synthesis method of EP toughening agent, which can effectively improve the compatibility between toughening agent and EP. Furthermore, the toughening mechanism is studied, which includes the curing reaction temperature, glass transition temperature, bonding strength, curing heating rate. Particularly, the amount of toughening agent is studied after EP system being toughened by the new synthesis toughening agent.

## Synthesis of toughening agent

### Materials

The materials are as follows:

1. Epoxy resin E54, Guangzhou Rong Sheng Chemical Co., Ltd.;
2. Polypropylene glycol (PPG), MW = 2000, Wanhua Chemical Group Co., Ltd.;
3. 4,4′-Diphenylmethane diisocyanate (MDI), MW = 250, Wanhua Chemical Group Co., Ltd.;
4. Polyethylene glycol (PEG), MW = 400, Wanhua Chemical Group Co., Ltd.;
5. Diethylenetriamine (DETA), curing agent, Changzhou Deye Chemical Industry Co., Ltd.

### Synthetic methods

Two thousand gram PPG, 250 g MDI and 400 g PEG were put into a three mouth flask, which is equipped with a mechanical stirrer, a reflux condenser and a thermometer. Then the three mouth flask was heated in oil and the blender was started, the hydroxyl-terminated group of PPG and PEG are connected by isocyanate functional group of MDI, which results in forming a hydroxyl-terminated polymer with ether bond and urethane bond. To synthesize the toughening agent, PPG, PEG and MDI were treated by vacuum dehydration, PPG and MDI are mixed and heated to 80 °C in the three mouth flask, then PEG is dropped into the three mouth flask and gradually heated up to 100 °C for 3 h. The chemical equation is shown as follows.
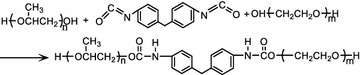


### Analysis of synthetic product

#### Molecular weight

The synthetic product is polydispersity, its molecular weight was obtained by the viscosity method (Frisch and Yeh 2003), which is based on MHS equation,

$$[\eta ] = K\overline{M}_{v}^{\alpha } .$$

After having measured the intrinsic viscosity of the polymer’s dilute solution, the viscosity- average molecular weight is calculated according to *α* and K. The synthetic toughening agent is dissolved and dubbed into different concentration of polymer solution, as shown in Table 1. After having measured the corresponding viscosity of different concentration polymer solution, the obtained viscosity-average molecular weight is 2563.5.

**Table 1:** Ionic concentration of different polymer solution

Sample no.	1 Pure solvent	2 Undiluted solution	3 Solution with 2 mL water	4 Solution with 3 mL water	5 Solution with 5 mL water	6 Solution with 10 mL water
Ionic concentration (10^−2^ mol/L)	0.1885	1.0112	0.8431	0.6745	0.5059	0.3372

#### Composition of toughening agent

Infrared absorption spectrum (Qi et al. 1988) generated by continual vibration and rotational motion of the molecules, the molecular vibrations means the relative motion of the atoms near the equilibrium position, polyatomic molecules can composite various vibration graphics. Infrared spectroscopy can be used to study the molecular structure and chemical bonds (Pandita et al. 2012), which can also be used as characteristically approach of chemical species. The functional group structure of the synthetic toughening agent was studied by using infrared absorption spectra.

**Fig. 1:**
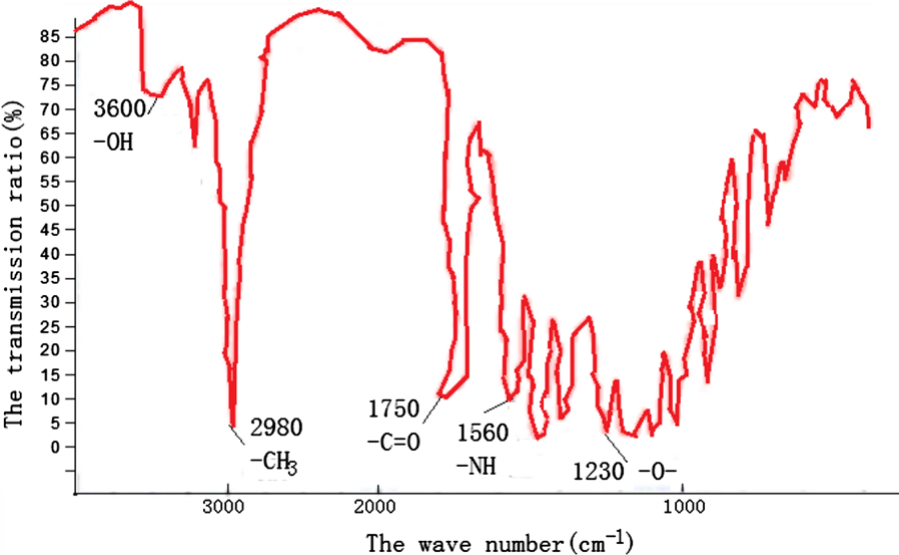
IR spectra of synthetic toughening agent

The experimental IR samples were produced by using KBr compressed tablet method, which need 1–2 mg toughening agent sample and 200 mg pure KBr cut into powder evenly, then they were placed in the mold and pressed into transparent sheets with a 5–10 MPa hydraulic machine. The infrared spectra of toughening agent is shown in Fig. 1, the vertical coordinate means the absorption intensity, and the horizontal coordinate means the wave number. Obviously, the molecular structure contains –OH, –C=O, –CH3, –NH, –O– functional groups according to the position of characteristic peaks.

#### Isocyanate group content

The molecular weight and functional group information of synthetic toughening agent can be obtained from the viscosity method and infrared absorption spectra, the experimental data and results are satisfactory. However, the chemical reaction is not simple, the involved substances are two prepolymer polyol molecules and small molecule chain extender. Due to differences in viscosity, the involved substances can not be completely in accordance with 1:1:1, the residual content of isocyanate group (–NCO), aelect toluene–dibutylamine system can be measured by using chemical titration (GB/T 12009.4-1989 1989). The method is as follows: in order to react with –NCO, the dibutylamine was dissolved in toluene, then the excessive dibutylamine is calibrated with standard HCl solution, consequently, the –NCO content of the sample can be obtained, the chemical equation is shown as follows.
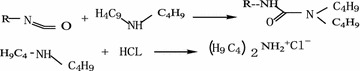


Bromocresol green was used as indicator during the titration process, while the color of bromocresol changes from green to yellow, which can record the volume of hydrochloric acid consumption. Then the −NCO content (Moghimi et al. 2014) was calculated according to the following formula, in which V_0_ and V_1_ mean the volume of hydrochloric acid consumption of the blank sample and the testing sample, c means concentration of hydrochloric acid standard solution, m means the quality of the sample. The residual isocyanate group ratio is 2.185 %, which comes from the experimental measured after polymerization.

$${\text{NCO}}\% = \frac{{\left( {{\text{V}}_{0} - {\text{V}}_{1} } \right) \times {\text{c}} \times 4. 2 0 2}}{\text{m}} = \frac{(32.41 - 31.89) \times 0.1 \times 4.202}{10} = 2.185\,\%$$

## Performance analysis of epoxy resin adhesive

The performance of synthetic toughening agent should be tested in EP adhesive system. Firstly, the adhesive system can be formed by adding a certain amount of toughening agent and curing agent into EP, and then some tests and experiments were performed to evaluate performance of the new synthetic toughening agent.

### Curing reaction temperature, curing heat and glass transition temperature

**Fig. 2:**
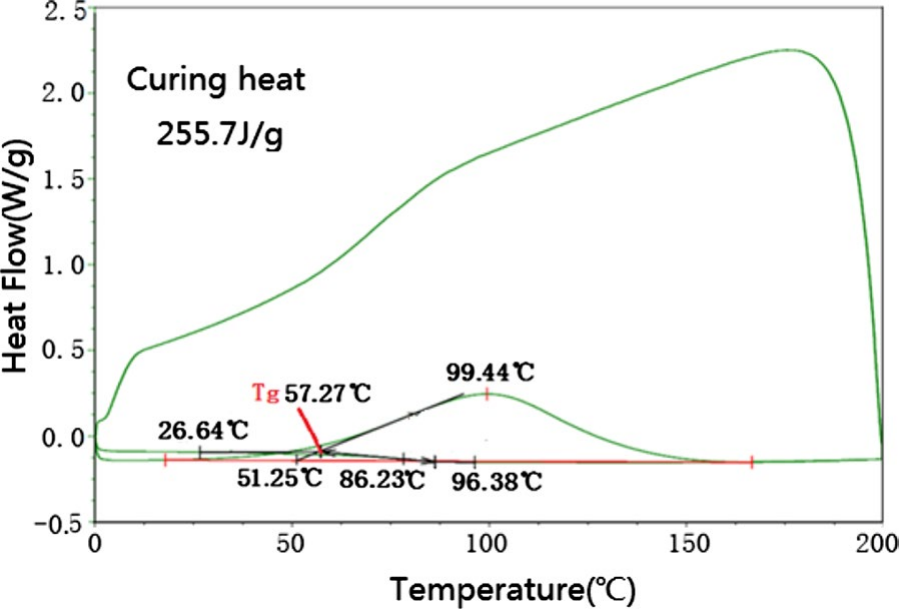
DSC diagram in 5 °C/min heating rate process

The differential scanning calorimetry (DSC) test had been done with pre-configured EP, the DSC curve of EP system after toughening is as shown in Fig. 2. The DSC test contains two heating process (Wang et al. 2013), the purpose of the first heating process is to accelerate curing for EP adhesive, the second heating process is to test the extent of curing reaction. On the first heating curve, the initial reaction temperature corresponds to the intersection point of tangent line of the maximum slope of curing reaction curve and the baseline (Kong et al. 2014), the integral area of curing exothermic peak is identified as the curing heat. For the sample, the curing heating rate, temperature range, curing temperature and curing reaction heat are 5 °C/min, 0–200 °C, 51.21 °C and 255.7 J/g respectively.

It can be seen from the second heating curve that there is no obvious crest, which indicates that resin has been cured completely after the first heating. The glass transition temperature (Tg) is regarded as the intersection point of the transition line’s extension line and the baseline’s extension line (Zhang et al. 2011), the glass transition temperature of the polymer is 57.27 °C. This experiment was performed at Physical and Chemical Test Center of the University of Science and Technology of China.

### The effects of heating rate on the curing temperature and curing heat

To reveal the effects of heating rate on the curing temperature and curing heat (Jang and Paik 2009), the DSC test of the same EP system in three different heating rates of 5, 10, 20 °C/min were performed respectively. The DSC curves of three different heating rate is drawn.

**Fig. 3:**
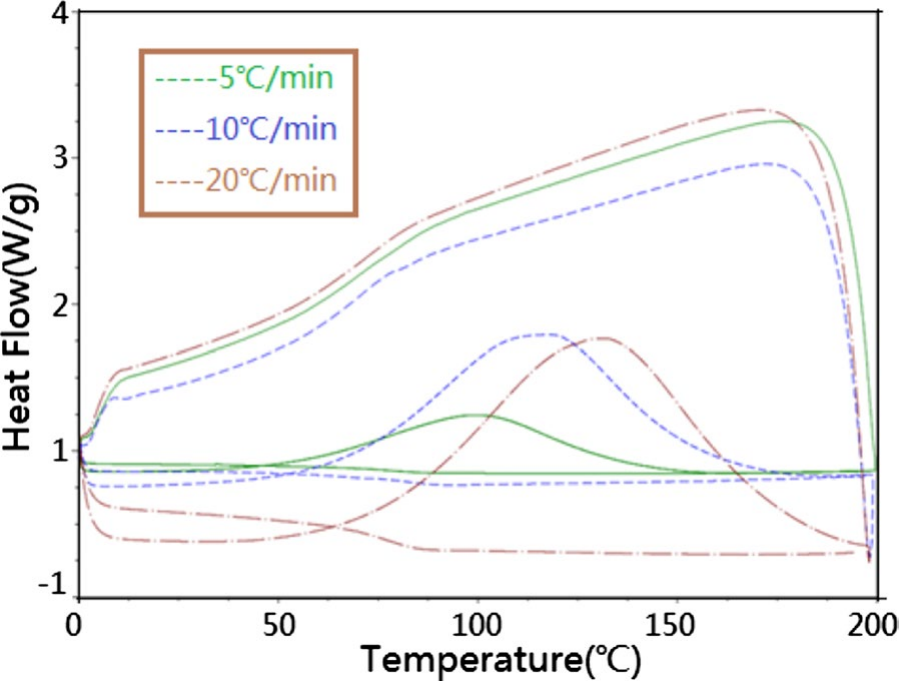
DSC diagram under three kinds of heating rate

**Fig. 4:**
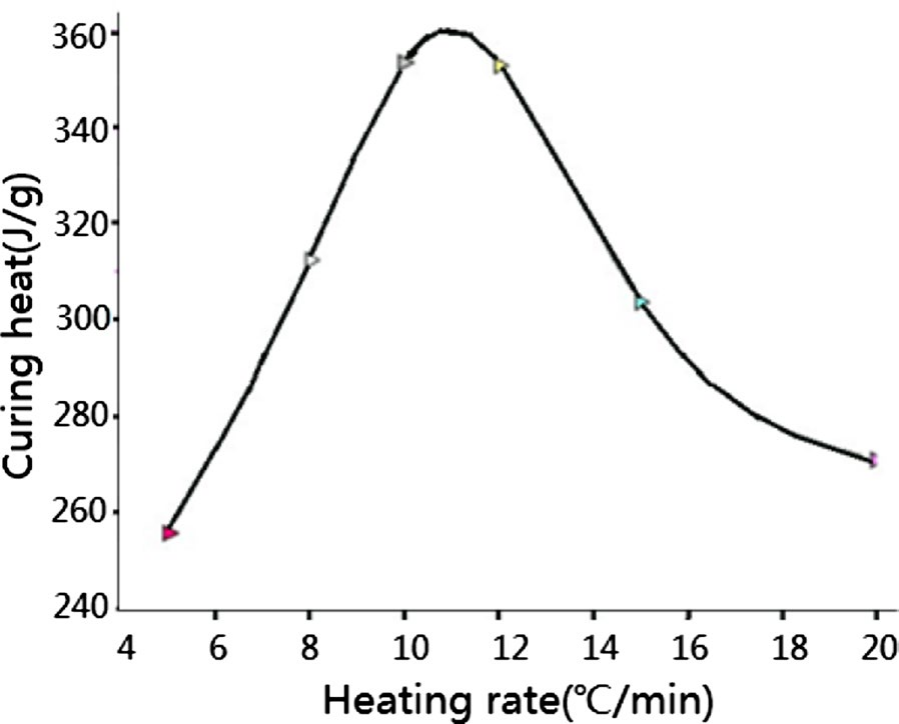
The curve of curing heat verse heating rate

In Fig. 3, it is cleared that the curing reaction temperature under three different heating rate are 51.21, 65.61, 74.24 °C respectively, which indicates that the curing reaction temperature of resin system increased with heating rate. The curing reaction heat under three cases are 255.7, 353.5, 271.0 J/g respectively, undoubtedly, the curing reaction heat is not always increased with the heating rate (Shanmugharaj and Ryu 2012). To obtain a comprehensive understanding of the specific relationship between the two physical quantities, another four groups of DSC tests under 6, 8, 12 and 20 °C/min heating rate had been done. The relationship between the curing heat and the heating rate is shown in Fig. 4. For the resin system, the peak curing heat locates in the middle part of the curve, which corresponds to the 11 °C/min heating rate.

### Microstructure of the specimen port

**Fig. 5:**
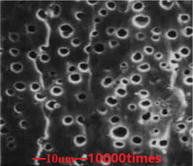
Formula A

**Fig. 6:**
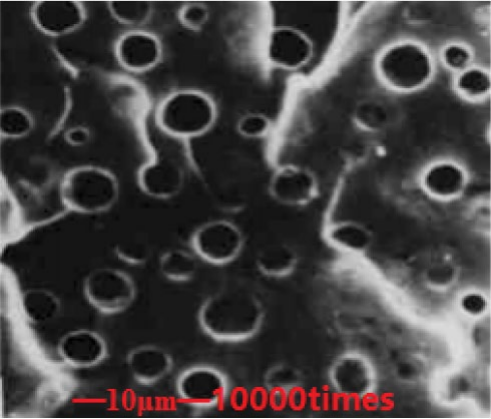
Formula B

**Fig. 7:**
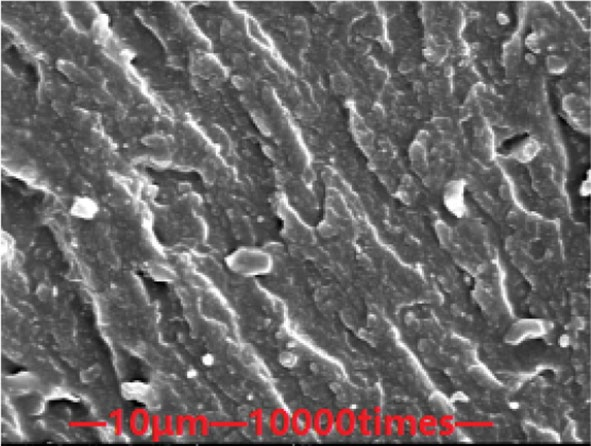
Formula C

To study the effect of toughened agent in the EP-curing agent system (Fan et al. 2013), the scanning electron microscope (SEM) was used to investigate the microstructure of the specimen port (Aguiar et al. 2012). Figures 5, 6 and 7 shows the microstructure of the specimen port of A formula, B formula and C formula, which correspond to the ratio of EP and toughening agent are four to one, five to one and no toughening agent respectively.

It can be seen from the SEM pictures that the shear port surface morphology of formulas A and B show two-phase structure, in which the continuous phase likes river and the dispersed phase likes holes. But the shear port surface morphology of formula C is randomly distributed. Formulas A and B both contain toughening agent, the content of toughening agent for formula B is higher, the hole density for formula B is more than formula A. While the EP-curing agent system constitutes the continuous phase, the new synthetic toughening agent distributes in it to form the dispersed phase. As a result, the two-phase structure greatly improves compatibility between the EP and additives.

### The bonding strength test

The bonding strength test of the toughened EP was performed on the calibrated MTS universal testing machine (Leal and Lima 2012). According to GB/T 13936-92, the dimensions of the specimen for bonding strength test are as follows:

1. The geometry size is 40 mm × 10 mm × 2 mm;
2. The overlap area is 40 mm × 10 mm.

Stainless steel sheets were used to clamp the specimen. After having filled the gap between two stainless steel sheets with epoxy adhesive, which was curing for 1 day at room temperature (24 °C), then the specimen with stainless steel sheets was installed on the testing machine to perform bonding strength test (Peles et al. 2004), the tensile rate was 2 mm/min. When the specimen was broken, the corresponding tensile force was 2.864 kN. According to formula (1):

1 $$\uptau = {\text{F}}/({\text{b}} \cdot {\text{l}})$$

**Fig. 8:**
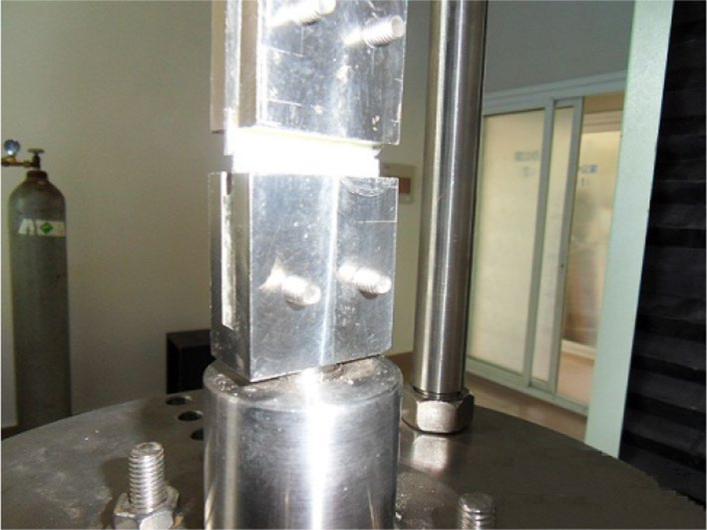
Tensile mold of material test machine

**Fig. 9:**
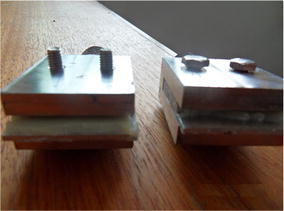
Adhesive pieces after fracturing

The test was done under room temperature (24 °C). The shear strength τ can be calculated by using formula (1). In formula (1), F means tensile force, b represents the width of specimen bonding surface, l means the length of specimen bonding surface. As a result, the bonding strength of the toughened EP is 35.8 MPa. Figures 8 and 9 shows the pictures of the tensile mold and the broken specimen respectively.

### Research on the effect of the toughening agent amount to the bonding strength

**Fig. 10:**
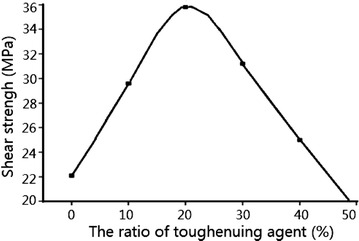
The curve of toughening agent’s amount and bonding strength

To study the relationship between the toughening agent amount and the bonding strength, the chosen ratio of EP adhesive to toughening agent is 0, 10, 20, 30, 4, 50 %, the obtained curve of toughening agent’s amount and bonding strength is as shown in Fig. 10.

When the ratio of EP adhesive to toughening agent is 20 %, the bonding strength reaches the peak value, however, if the ratio of toughening agent is too high, which will cause damage to the original curing system, thus reducing the system’s bonding strength (Al-Turaif 2010). If the ratio of toughening agent is <20 %, a perfect two-phase structure was formed by the combination of toughening agent and resin system without affecting the epoxy value of the system, therefore, the bonding strength of the system can be improved.

## Conclusions

1. The compatibility between the synthesized toughening agent and EP E54 is satisfactory.
2. The epoxy resin E54 system can be cured completely under proper condition, the curing heat reaches the peak value at the heating rate of 11 °C/min, therefore, it is necessary to select the proper heating rate.
3. In the epoxy resin E54 system toughened by the synthesized toughening agent, toughening agent contributes to form a perfect two-phase structure. When the ratio of curing agent and epoxy resin is fixed, the proportion of toughening agent affects the bonding strength of epoxy resin system greatly.
